# Energy status and HIF signalling in chorionic villi show no evidence of hypoxic stress during human early placental development

**DOI:** 10.1093/molehr/gau105

**Published:** 2014-11-11

**Authors:** T. Cindrova-Davies, M. Tissot van Patot, L. Gardner, E. Jauniaux, G. J. Burton, D. S. Charnock-Jones

**Affiliations:** 1 Centre for Trophoblast Research, Department of Physiology, Development and Neuroscience, University of Cambridge, Cambridge CB2 3EG, UK; 2 Academic Department of Obstetrics and Gynaecology, Royal Free and University College, London, UK; 3 Department of Obstetrics and Gynaecology, University of Cambridge, Cambridge CB2 0SW, UK; 4 National Institute for Health Research, Cambridge Comprehensive Biomedical Research Centre, Cambridge, UK

**Keywords:** placenta, HIF-1α, first trimester, metabolomics, energy status

## Abstract

Early human placental and embryonic development occurs in a physiologically low oxygen environment supported by histiotrophic secretions from endometrial glands. In this study, we compare the placental metabolomic profile in the first, second and third trimesters to determine whether the energy demands are adequately met in the first trimester. We investigated whether hypoxia-inducible factors, HIF-1α and/or HIF-2α, might regulate transcription during the first trimester. First and second trimester tissue was collected using a chorionic villus sampling-like (CVS) technique. Part of each villus sample was frozen immediately and the remainder cultured under 2 or 21% O_2_ ± 1 mM H_2_O_2,_ and ±the p38 MAPK pathway inhibitor, PD169316. Levels of HIF-1α were assessed by western blotting and *VEGFA*, *PlGF* and *GLUT3* transcripts were quantified by RT–PCR. Term samples were collected from normal elective Caesarean deliveries. There were no significant differences in concentrations of ADP, NAD^+^, lactate, and glucose, and in the ATP/ADP ratio, across gestational age. Neither HIF-1α nor HIF-2α could be detected in time-zero CVS samples. However, culture under any condition (2 or 21% O_2_ ± 1 mM H_2_O_2_) increased HIF-1α and HIF-2α. HIF-1α and HIF-2α were additionally detected in specimens retrieved after curettage. HIF-1α stabilization was accompanied by significant increases in *VEGFA* and *GLUT3* and a decrease in *PlGF* mRNAs. These effects were suppressed by PD169316. In conclusion, our data suggest that first trimester placental tissues are not energetically compromised, and that HIF-1α is unlikely to play an appreciable role in regulating transcriptional activity under steady-state conditions *in vivo*. However, the pathway may be activated by stress conditions.

## Introduction

The human placenta differentiates in a physiologically low oxygen environment, supported by histiotrophic secretions from the endometrial glands (Hempstock *et al.*, 2004). A major change takes place at the end of the first trimester when the maternal arterial circulation commences (Burton *et al.*, 1999), and the oxygen tension within the intervillous space increases from ∼18 mmHg (equivalent to 2.5% O_2_) at 8 weeks to ∼60 mmHg (8.5%) at 12 weeks (Jauniaux *et al.*, 2000, 2003). This rise is associated with a burst of oxidative stress in the placental tissues, and with a robust increase in antioxidant defences (Burton and Jauniaux, 2004).

The low oxygen environment is essential for normal embryonic and placental development, and premature onset of blood flow can contribute to pregnancy failure (Hustin *et al.*, 1990; Jauniaux *et al.*, 2003). Metabolism appears adapted to these conditions, for we have shown that polyol pathways are highly active in first trimester placental tissues (Jauniaux *et al.*, 2005). These pathways provide an important mechanism for the reoxidation of pyridine nucleotides under conditions of low oxygenation, enabling glycolysis to continue without an excessive rise in acidity. We speculate that the metabolic needs of the first trimester conceptus are adequately met under 2.5% O_2_ through these pathways. To test this hypothesis, we here compare the metabolomic profile of first trimester (7–8 weeks), second trimester (14–16 weeks) and term placental samples.

A variety of genes are regulated by oxygen, including vascular growth factor (*VEGFA*), glycolytic enzymes, erythropoietin (*EPO*), haemoxygenase-1 (*HO1*) and inducible nitric oxide synthetase (*iNOS*). Under hypoxic conditions, activation of these genes maintains the oxygen supply and energy balance by inducing vasodilation, erythropoiesis, angiogenesis and energy production. The hypoxia-inducible factor (HIF) family of basic Helix–Loop–Helix transcription factors plays a key role in inducing these adaptive responses in other systems (Semenza *et al.*, 1994; Forsythe *et al.*, 1996; Semenza, 1999, 2001; Giaccia *et al.*, 2003; Pugh and Ratcliffe, 2003). HIF-1α and HIF-2α exhibit high sequence homology, and activate common as well as unique target genes. HIF-3α lacks a transcriptional activation domain and represses O_2_-regulated gene expression (Pouyssegur and Mechta-Grigoriou, 2006). HIF-1α heterodimerises with the constitutively expressed HIF-1β (ARNT) and binds to a short DNA motif identified in the 5′-flanking regions of many hypoxia-induced genes (Wang *et al.*, 1995). HIF-1α is rapidly degraded under normoxic conditions through interaction with von Hippel–Lindau protein (VHL) (Maxwell *et al.*, 1999; Bruick and McKnight, 2001; Ivan *et al.*, 2001). HIF-1α stability is increased by hypoxia, but can also be enhanced under non-hypoxic conditions by inflammatory cytokines or microtubule-depolymerizing agents involving the NF-κB pathway (Jung *et al.*, 2003a, b, c). Thus, HIFs might not only regulate the effects of changing O_2_ supply on placental development, but also the effects of hormones, growth factors and cytokines (Pringle *et al.*, 2010; Kuschel *et al.*, 2011).

Both HIF-1α and HIF-2α proteins are constitutively expressed in the human placenta. HIF-2α mRNA increases significantly with gestational age, whereas HIF-1α mRNA is expressed at a constant level in all placentas but with greater variability in early pregnancy (Rajakumar and Conrad, 2000). In contrast, the abundance of both HIF-1α and HIF-2α protein has been reported to decrease significantly with gestational age (Caniggia *et al.*, 2000; Rajakumar and Conrad, 2000). Two peaks in HIF-1α mRNA and protein seem to occur during early development at 7–10 and 14–18 weeks (Ietta *et al.*, 2006). As the intervillous pO_2_ rises at 12–14 weeks, the second peak in HIF-1 expression is likely to be induced by factors other than O_2_, possibly oxidative stress-induced pro-inflammatory cytokines (Ietta *et al.*, 2006). The role of oxygen in regulating the first peak is also questionable, since HIF signalling typically shows maximal activity between 0.5 and 2% O_2_ (Gorr *et al.*, 2010), whereas the tension in the first trimester corresponds to ∼2.5% O_2_. Conflicting results have been reported regarding the placental response to hypoxia *in vitro*. Rajakumar and Conrad (2000) cultured first trimester and term villus explants under 2 or 21% O_2_ for 3–24 h and reported increases in HIF-1α and HIF-2α protein and HIF-1α DNA binding activity in the 2% O_2_ samples, but no changes in their mRNA levels. In contrast, Caniggia *et al.* (2000) cultured first trimester explants at 3 or 21% O_2_ for 24 h and reported an increase in both HIF-1α protein and mRNA under reduced oxygen. Furthermore, Genbacev *et al.* (2001) reported an increase only in HIF-2α in first trimester explants following 48 h incubation under 2% O_2_. Given the more recent data that HIFs can be stabilized by oxygen-independent pathways, we speculate that these changes in HIF abundance might have been influenced by the tissue collection procedure. Early placental samples are usually obtained by curettage, when they are inevitably exposed artifactually to maternal blood. For samples of <10 weeks gestational age, this represents a potential stressor. Hence, in this study, we studied HIF-1α and HIF-2α in samples collected using a chorionic villus sampling-like (CVS) technique under ultrasound guidance; these samples were free of maternal blood contamination. We compared data from these samples with those obtained using material collected by curettage. We also studied HIF-1α protein abundance in short-term-cultured first trimester CVS explants under 2% O_2_ and 21% O_2_, in order to assess the functional significance of changes in HIF-1α in terms of its downstream gene targets.

## Materials and Methods

### Sample collection

First trimester placental samples were collected with written informed consent and approval from the University College London Hospitals Committee on the Ethics of Human Research or the Cambridge Local Research Ethics Committee from patients undergoing surgical termination of normal pregnancies. The method of sample collection varied depending on the purpose of study, i.e. first trimester explant culture, metabolomics study or comparison of different collection methods. Our first and second trimester tissues are routinely collected using a CVS technique under ultrasound guidance from the central region of the placenta. Archival paraffin blocks were used for additional immunostaining, and frozen samples were used for the metabolomics study. For the first trimester explant cultures, we obtained tissue from 15 cases ranging from 5 to 12 weeks gestational age. Gestational age was estimated from the crown rump length of the fetus. Part of the sample was frozen immediately (<2 min) in liquid nitrogen (time zero, *T*_0_), part was fixed in 4% paraformaldehyde and embedded in paraffin wax for immunohistochemistry, and part was collected into medium equilibrated with 2% O_2_/5% CO_2_ and transported on ice.

To assess the effect of stress introduced as a collection artefact, we also obtained 4 first trimester frozen and 10 paraffin wax-embedded samples retrieved from suction curettage bags. All samples were anonymized, and were from normal pregnancies.

For the metabolomics study, we collected five first trimester samples (7–8 weeks), five second trimester samples (14–16 weeks) under ultrasound guidance by CVS as described above, and four term samples within 10 min of delivery by Caesarean section from healthy pregnancies (Serkova *et al.*, 2003).

### Tissue culture

First trimester villus explants were cultured under 2 or 21% O_2_ for 6 h ± 1 mM H_2_O_2_. In each combination, the p38 MAPK pathway inhibitor PD169316 was used at 10 µM, as previously optimised (Cindrova-Davies *et al.*, 2007a). Following culture, the samples were snap-frozen for western blotting and RNA extraction. Transcript levels for the target genes *VEGFA, PlGF* and *GLUT3* were quantified by quantitative real-time RT–PCR.

### Western blots

Tissue homogenization and SDS–PAGE electrophoresis and immunoblotting were carried out as previously described (Tjoa *et al.*, 2006). Primary antibodies against phospho-p38, phospho-AKT, TNF-α, Hsp27 and IκB were from Cell Signaling (all used at 1:1000; New England Biolabs, Hitchin, UK); anti-HIF-1α was from Novus Biologicals (used at 1:500; Cambridge, UK). Proteins were revealed and quantified using Image J software (National Institutes of Health, http://rsb.info.nih.gov/ij/). Protein loading was normalized against β-actin or Poncaeu S staining. Values are expressed as a percentage of the control lysate (100%) for each experiment. Western blot measurements were analysed using ANOVA, and the Protected Least Significant Differences *post hoc* test (Fisher's *post hoc* test). Differences between two groups were evaluated using a Fisher's *post hoc* test. Results were considered significant at *P* < 0.05.

### Immunohistochemistry

Immunohistochemistry with DAB detection was performed according to a protocol described previously (Tjoa *et al.*, 2006). Anti-HIF-1α was from Novus Biologicals (used at 1:250; Cambridge, UK) and anti-HIF-2α was from Abcam (used at 1:250; Cambridge, UK). Both antibodies required heat-induced antigen retrieval (Tris-EDTA buffer, pH 9).

### Immunoprecipitation

To isolate phosphorylated proteins, villous protein lysate (250 µg) was pre-cleared using A/G PLUS-Agarose Immunoprecipitation Reagent (Santa Cruz, CA, USA) at 4°C for 30 min. Anti-phospho-Ser/Thr/Tyr antibody (Santa Cruz) was added (1 µg) and incubated at 4°C overnight. Protein A/G PLUS-Agarose beads were added, incubated at 4°C for 6 h, and recovered and washed four times. After the final wash, pellets were resuspended in 15–20 µl 3× SDS–PAGE buffer, boiled for 3–5 min and analysed by western blotting.

### Sample preparation for ^1^H- and ^31^P-NMR spectroscopy

Placental tissues were extracted using 8% perchloric acid (Sigma-Aldrich Co., St Louis, MO, USA) as previously described (Tissot van Patot *et al.*, 2010). Briefly, perchloric acid was added to powdered tissues and centrifuged (20 min, 1300*g*, 4°C). Hydrophilic metabolites (supernatant) were collected and the procedure repeated on the pellet. The supernatants were combined, neutralized (KOH), centrifuged to remove potassium perchlorate, lyophilized and dissolved in deuterium oxide (D_2_O) for NMR analysis. Lipophilic metabolites (pellet) were neutralized, lyophilized and dissolved in 0.6 ml of deuterated chloroform/methanol mixture (2:1, vol/vol) for ^1^H-NMR Analysis.

### Quantitative ^1^H-NMR analysis

Hydrophilic extracts were analysed by high-resolution ^1^H- and ^31^P-NMR using a 500 MHz high-resolution Bruker DRX system equipped with Bruker TopSpin software (Bruker Biospin Inc., Fremont, CA, USA) (Serkova *et al.*, 2007). An inverse TXI 5-mm probe was used for all ^1^H-NMR experiments. An external reference, trimethylsilyl propionic-2,2,3,3,-d4 acid, was used for metabolite quantification of fully relaxed ^1^H-NMR spectra and as a ^1^H chemical shift reference (0 ppm). A two-dimensional (2D)-^1^H, ^13^C-HSQC (heteronuclear single quantum correlation) NMR sequence was used for metabolite identification. The ^1^H-NMR peaks for single metabolites were identified and referred to a metabolite chemical shift library. After performing Fourier transformation and making phase and baseline corrections, each ^1^H peak was integrated using 1D WINNMR (Bruker Biospin Inc.). The absolute concentrations of single metabolites were then referred to the TMSP integral.

The water-soluble (hydrophilic) placental extracts were additionally analysed by ^31^P-NMR spectroscopy immediately after ^1^H-NMR (EDTA was to chelate divalent ions bound to ATP) (Serkova *et al.*, 2007). Phosphorous spectra were obtained on a Bruker 300 MHz Avance spectrometer (^31^P-NMR frequency: 121.5 MHz) equipped with a 5-mm QNP ^31^P/^13^C/^19^F/^1^H probe using a composite pulse-decoupling (CPD) program. An external standard in a thin capillary, methyl diphosphoric acid (MDP, 2.3 mmol/l D_2_O, Sigma-Aldrich), was placed into the NMR tube to serve as a reference for both chemical shift (18.6 ppm) and phosphor metabolite quantification.

Data were tested using a non-parametric Kruskal–Wallis test, with *P* < 0.05 being considered significant.

### Creatine kinase activity assay

Creatine kinase (CK) activity was measured in five first trimester (6–7 weeks), five second trimester (14 weeks) and five term samples (39 weeks), according to the manufacturer's instructions (Abcam, Cambridge, UK).

### RNA isolation and quantitative real-time RT–PCR analysis

Total RNA was isolated from snap-frozen placental tissue using RNAeasy kit (Qiagen, Crawley, UK). RNA was quantified by spectrophotometry (Nanodrop Technologies, DE, USA) and integrity was assessed using an Agilent 2100 bioanalyser (Agilent Technologies UK Limited, UK). In brief, 20 µg of total RNA from each sample was reverse transcribed using a master mix containing SuperScript II Reverse Transcriptase in the First Strand Buffer with 0.1 M DTT (Invitrogen, Paisley, UK) and 50 ng/ml random hexamers (Sigma). The ABI PRISM 7700 Sequence Detection System (TaqMan) was used to perform real-time PCR according to the manufacturer's protocols. Ct values for each transcript were compared with those for 18S rRNA (dCt obtained), and these values were compared with *T*_0_ samples (ddCt values are reported). Primers for *VEGFA* (Hs00173626-m1), *PlGF* (Hs00182176-m1), *GLUT3* (Hs00359840-m1) and 18S (Hs99999901-s1) were obtained from Applied Biosystems (ABI, Warrington, UK).

## Results

### Energy status of first trimester, second trimester and term placental tissue

**Figure 1:**
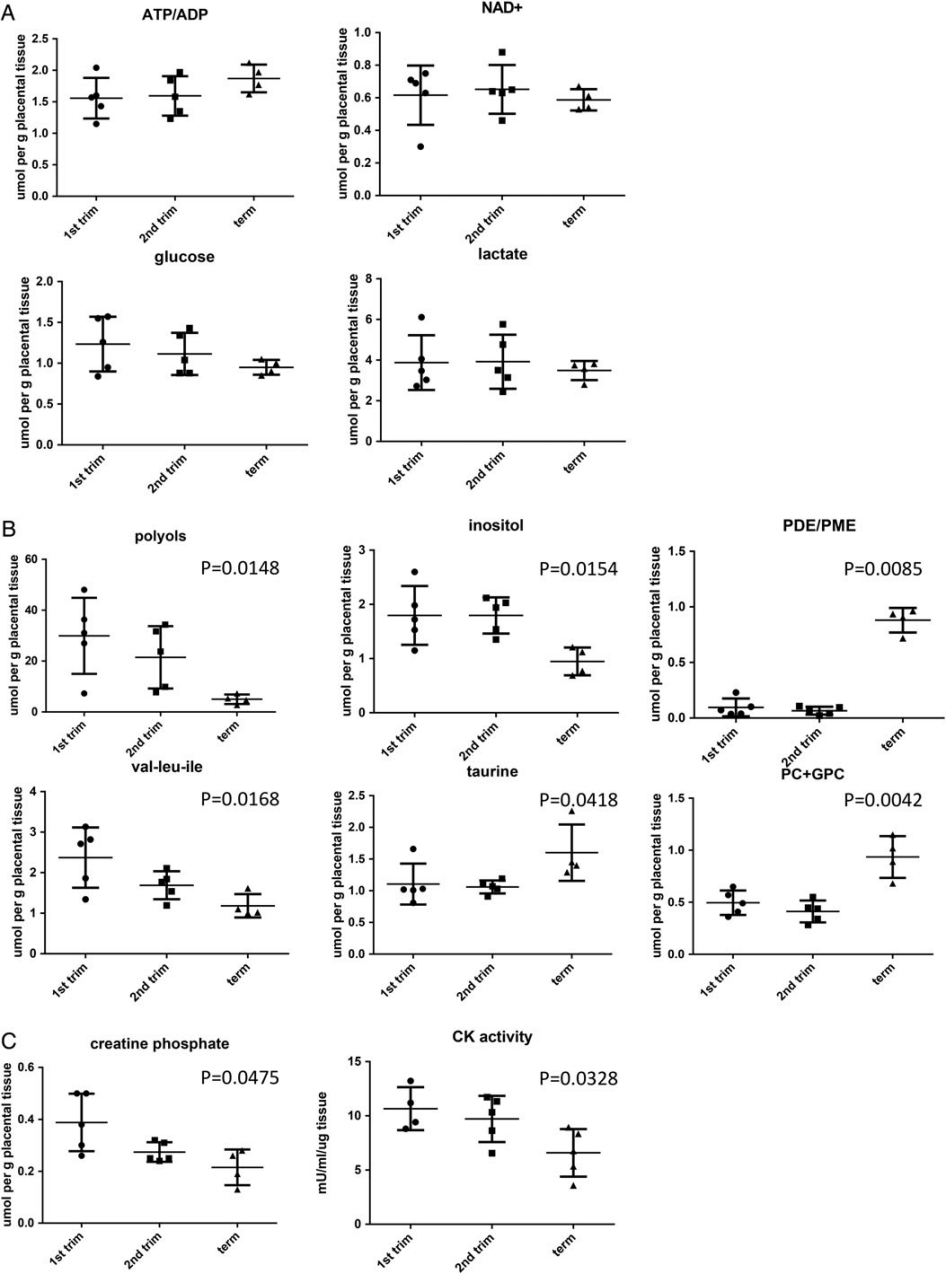
The metabolomic profile of first (*n* = 5) and second trimester (*n* = 5) and term placental tissue (*n* = 4). Tissue samples were subjected to quantitative ^1^H-NMR analysis to measure the expression of individual metabolites (expressed as µmol per g placental tissue). (**A**) There were no significant differences in ATP/ADP ratio, or expression of NAD^+^, lactate or glucose among the groups. (**B**) The concentrations of taurine increased significantly with increased gestation, whilst concentrations of polyols, inositol and hydrophobic amino acids (val/leu/ile) decreased with advanced gestation. (**C**) Creatine phosphate concentration (measured by NMR) was significantly decreased with increased gestational age and was confirmed by reduced CK activity measured placental homogenates by ELISA. All results were analysed by a non-parametric Kruskal–Wallis test.

There were no significant differences in the concentrations of the main energy metabolites, such as ADP, NAD^+^, lactate and glucose, between first and second trimester samples (Table I and Fig. 1A). Importantly, there was no difference in the ATP/ADP ratio, confirming that the first trimester placenta has sufficient ATP and energy reserves to fulfil its energy needs and support the growing conceptus (Fig. 1A).

**Table I:** Metabolite concentrations in placental tissue samples.

Parameter	7–8 weeks (*n* = 5)	14–16 weeks (*n* = 5)	Term (*n* = 4)
Acetate	0.118 (0.07)	0.134 (0.077)	0.065 (0.042)
ADP	0.466 (0.063)	0.6 (0.096)	0.325 (0.058)
Alanine	0.622 (0.047)	0.596 (0.116)	0.448 (0.023)
AMP	0.404 (0.064)	0.316 (0.064)	0.2 (0.081)
Arginine	0.336 (0.05)	0.266 (0.039)	0.208 (0.036)
Aspartate	0.178 (0.028)	0.224 (0.026)	0.295 (0.034)
ATP	0.742 (0.155)	0.938 (0.168)	0.59 (0.078)
ATP/ADP	1.56 (0.144)	1.6 (0.14)	1.87 (0.11)
Cholesterol	1.61 (0.287)	1.79 (0.164)	2.37 (0.057)
Choline (H_2_O soluble fraction)	0.312 (0.055)	0.320 (0.059)	0.178 (0.045)
Cholines (lipid fraction)	3.0 (0.493)	3.79 (0.109)	3.39 (0.186)
Creatine phosphate	**0.388 (0.05)**	**0.274 (0.017)**	**0.215 (0.034)**^a^
Glucose	1.234 (0.15)	1.114 (0.115)	0.95 (0.046)
Glutamate	1.202 (0.143)	1.336 (0.151)	1.01 (0.044)
Glutamine	0.546 (0.074)	0.59 (0.089)	0.345 (0.043)
Glycerol phosphate	1.696 (0.27)	1.848 (0.121)	1.7 (0.133)
Glutathione	0.308 (0.108)	0.36 (0.118)	0.43 (0.088)
Hydroxybuterate	0.062 (0.012)	0.072 (0.024)	0.12 (0.036)
Inositol	**1.796 (0.242)**	**1.796 (0.149)**	**0.95 (0.128)**^a^
Lactate	3.878 (0.602)	3.92 (0.594)	3.49 (0.235)
MUFA	6.516 (1.748)	6.718 (1.462)	5.45 (0.519)
NAD+	0.616 (0.081)	0.652 (0.067)	0.59 (0.033)
Pcho + GPC	**0.496 (0.052)**	**0.412 (0.047)**	**0.935 (0.1)**^a^
Phophatidyl choline	0.588 (0.085)	0.724 (0.012)	0.628 (0.038)
PDE	**0.550 (0.186)**	**0.380 (0.141)**	**2.8 (0.337)**^a^
PME	6.102 (0.679)	5.658 (1.161)	3.17 (0.274)
Phosphatidylethanolamine	0.158 (0.037)	0.248 (0.011)	0.165 (0.015)
PDE/PME	**0.095 (0.032)**	**0.066 (0.014)**	**0.88 (0.048)**^a^
Polyols	**29.936 (6.680)**	**21.478 (5.469)**	**5.0 (0.94)**^a^
PUFA	10.42 (1.722)	13.326 (0.578)	10.42 (0.756)
PUFA/MUFA	1.942 (0.343)	2.340 (0.447)	1.95 (0.187)
Succinate	0.350 (0.082)	0.346 (0.029)	0.29 (0.033)
TAG	0.868 (0.087)	1.042 (0.113)	0.705 (0.0378)
Taurine	**1.106 (0.144)**	**1.06 (0.046)**	**1.6 (0.223)**^a^
Total FA	34.58 (5.83)	40.32 (2.27)	44.78 (1.217)
UDPG	0.272 (0.037)	0.338 (0.026)	0.242 (0.054)
Val, leu, Ile	**2.372 (0.333)**	**1.69 (0.155)**	**1.18 (0.144)**^a^

^a^Significantly different *P* < 0.05 (non-parametric one-way ANOVA plus Kruskal–Wallis test). Numbers in bold are significantly different between the groups.

The data also indicate that the concentrations of phosphodiesters (PDE), phosphocholine (Pcho) and glycerolphosphocholine (GPC), and taurine increase during gestation, whilst those of polyols, inositol, creatine phosphate and hydrophobic amino acids (val/leu/ile) decrease (Table I, Fig. 1B). We validated the decrease in creatine phosphate by measuring the enzymatic activity of CK, and found the CK activity to be significantly reduced in term samples compared with the first or second trimester placentas (Fig. 1C).

### HIF-1α and HIF-2 α in first trimester placenta

**Figure 2:**
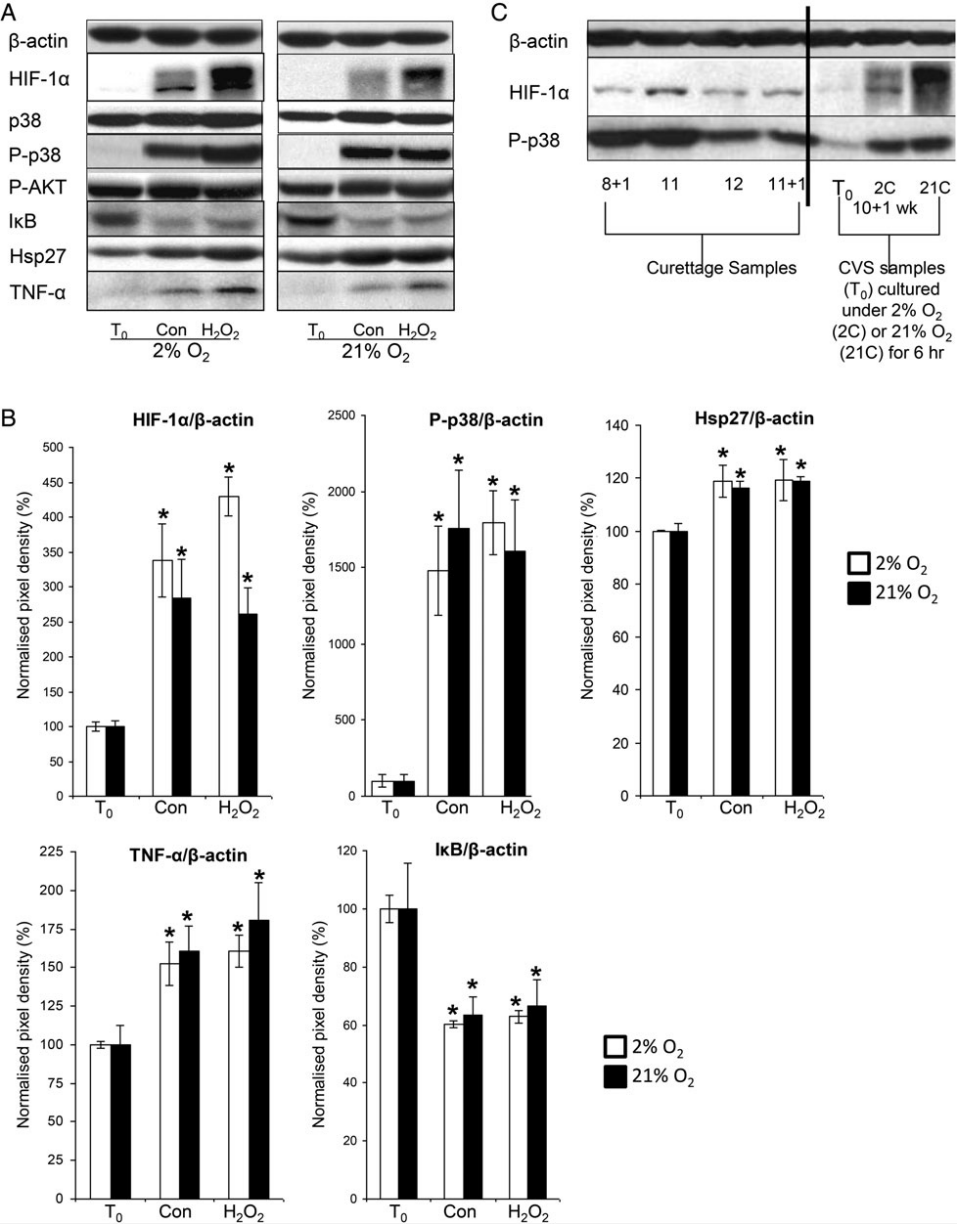
HIF-1α, p-p38, p38, Hsp27, TNF-α, IκB and P-Akt in first trimester samples (*n* = 6) cultured under 2% (white bars) or 21% O_2_ (black bars) in the presence or absence of 1 mM H_2_O_2_ for 6 h. (**A**) Lysates from first trimester explants were immunoblotted with antibodies against HIF-1α, p-p38, p38, Hsp27, TNF-α, IκB and P-AKT and (**B**) quantified by densitometry. (**C**) Lysates from representative first trimester curettage samples and a representative CVS sample cultured under 2% O_2_ (2C) or 21% O_2_ (21C) were immunoblotted with antibodies against HIF-1α, or phospho-p38. β-Actin staining served to normalize gel loading. Normalized results (±SEM) are plotted, expressing *T*_0_ samples as 100%. Significant differences (*P* < 0.05) are: * versus T_0_ samples (one-way ANOVA + Student–Newman–Keuls test). Con—denotes samples cultured under a given oxygen concentration for 6 h.

HIF1-α was virtually undetectable by western blotting in first trimester placental tissue collected by CVS. Culture of this tissue under 2 or 21% oxygen induced a marked increase in HIF-1α compared with *T*_0_ samples, and administration of the pro-oxidant H_2_O_2_ had no additional significant effect at either oxygen level (Fig. 2A). Culture at 2 or 21% O_2_ induced an increase in phosphorylation of the stress-induced mitogen-activated protein kinase p38 compared with *T*_0_ levels, but had no effect on the total level of p38 or AKT phosphorylation (Fig. 2A and B). Additionally, there was evidence of increased inflammatory markers in cultured tissue, as demonstrated by increased TNF-α, reduced IκB and increased Hsp27. Addition of 1 mM H_2_O_2_ had no additional effect on any of these markers (Fig. 2A and B).

In contrast, high levels of HIF-1α were apparent in first trimester samples obtained by curettage (Fig. 2C). Multiple HIF-1α bands were apparent in some samples, indicative of phosphorylation (Fig. 2A and C). In addition to increased HIF-1α, there was a marked increase in the phosphorylation of the stress kinase p38 detectable in curettage samples, as in the cultured samples (Fig. 2C).

**Figure 3:**
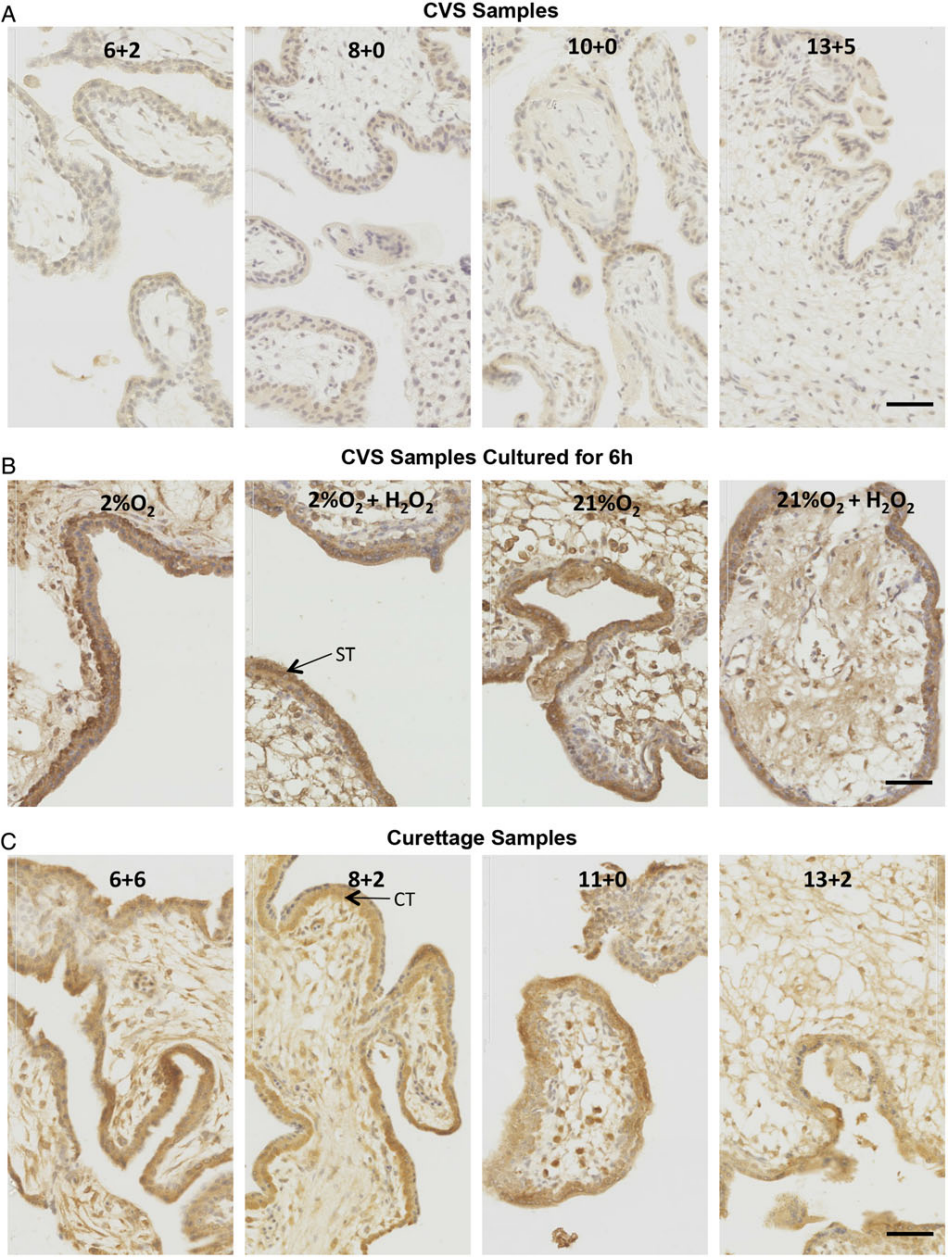
HIF-1α localization in first trimester placentas collected by a CVS-like technique (**A**), collected by CVS and then cultured for 6 h (**B**) or collected by curettage (**C**). Representative images of HIF-1α staining are shown. Gestational age is indicated in each representative image in (A and B), and culture conditions are indicated in (C). Brown colour signifies positive staining. HIF-1α was almost undetectable in the CVS tissue (A) whilst a prominent cytotrophoblast and syncytiotrophoblast nuclear staining was detected in all curettage specimens (B) and in cultured explants (C). Scale bar = 50 µm. ST, arrow points to a syncytiotrophoblast nucleus; CT, arrow points to a cytotrophoblast nucleus.

**Figure 4:**
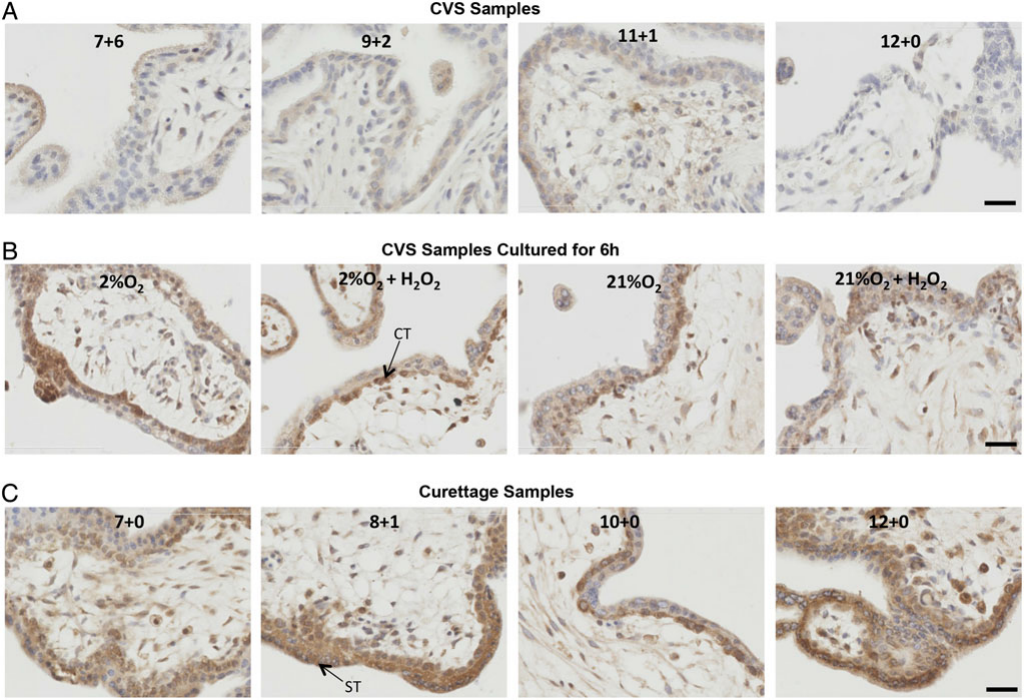
HIF-2α localization in first trimester placentas collected by a CVS-like technique (**A**), collected by CVS and then cultured for 6 h (**B**), or collected by curettage (**C**). Representative images of HIF-2α staining are shown. Gestational age is indicated in each representative image in A and B, and culture conditions are indicated in C. Brown colour signifies positive staining. HIF-2α was almost undetectable in the CVS tissue (A) whilst a prominent cytotrophoblast and syncytiotrophoblast nuclear staining was detected in all curettage specimens (B) and in cultured explants (C). Scale bar = 50 µm. ST, arrow points to a syncytiotrophoblast nucleus; CT, arrow points to a cytotrophoblast nucleus.

We confirmed the changes in HIF-1α by immunostaining. Whilst first trimester samples of various gestational ages collected by the CVS-like method showed minimal staining, and virtually no nuclear staining suggestive of transcriptional activity (Fig. 3A), there was marked nuclear localization of HIF-1α in all first trimester explants cultured for 6 h under 2 or 21% O_2_ in the presence or absence of H_2_O_2_ (Fig. 3B). Gestationally matched first trimester samples obtained by curettage (Fig. 3C) showed a similar staining pattern. The nuclear staining of explant and curettage samples localized primarily in syncytiotrophoblast and cytotrophoblast nuclei (Fig. 3B and C). There was no change in the pattern or intensity of staining with respect to gestational ages between 6 and 13 weeks. These results suggest that HIF-1α can be induced by stress. Similarly, we also examined HIF-2α in these groups of samples and found a strikingly similar pattern of staining. There was minimal nuclear staining in first trimester samples of various gestational ages collected by the CVS-like method (Fig. 4A), whilst there was marked nuclear localization of HIF-2α in all first trimester explants cultured for 6 h under 2 or 21% O_2_ ± H_2_O_2_ (Fig. 4B). There was also marked nuclear localization in gestationally matched first trimester samples obtained by curettage (Fig. 4C), suggesting that HIF-2α can also be induced by stress. Since HIF-2α is not involved in the regulation of VEGF in bone marrow, playing only an indirect role in haematopoiesis through small changes in the microenvironment (Scortegagna *et al.*, 2003), we decided to concentrate on the regulation and functional activity of HIF-1α in subsequent experiments.

### Increased *VEGFA* and decreased *PlGF* mRNA in cultured first trimester explants

**Figure 5:**
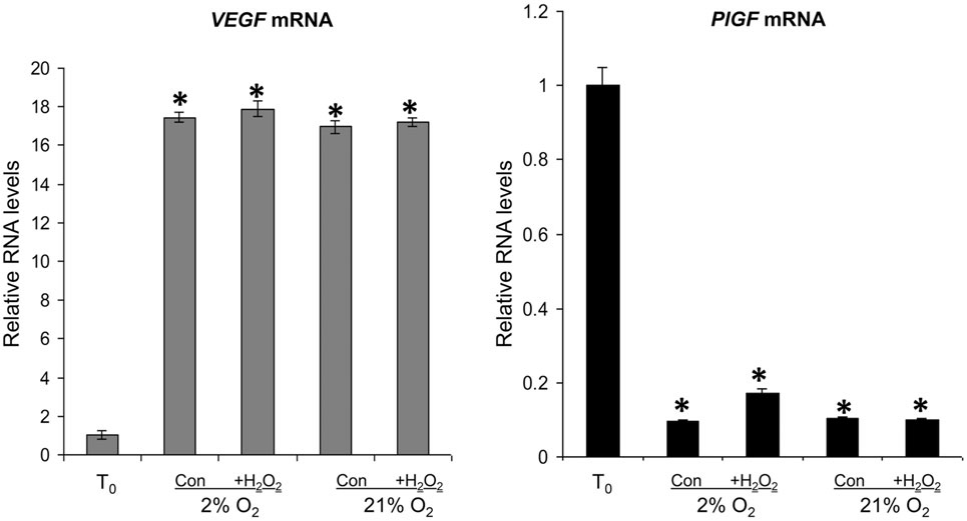
*VEGFA* and *PlGF* mRNA in *T*_0_ controls (*T*_0_), and in explants cultured under 2 or 21% O_2_ ± 1 mM H_2_O_2_ for 6 h. RNA was isolated and relative levels of *VEGFA* and *PlGF* mRNA were detected using quantitative real-time RT–PCR. *VEGFA* and *PlGF* mRNA levels were normalized to the 18S RNA levels. Significant differences (*P* < 0.05) are: * versus *T*_0_ controls (one-way ANOVA + Student–Newman–Keuls test).

To investigate whether the increase in HIF-1α was of functional significance, we measured the mRNA levels of *VEGFA* and *PlGF* by quantitative real-time RT–PCR in the *T*_0_ samples, and after culture in 2% O_2_ or 21% O_2_ ± 1 mM H_2_O_2_ for 6 h. All cultured samples showed a significant increase in *VEGFA* mRNA and significant decrease in *PlGF* mRNA, compared with *T*_0_ values (Fig. 5). Administration of H_2_O_2_ had no additional effect.

### Changes in HIF-1α protein and *VEGFA* and *GLUT3* mRNA in first trimester explants with p38 inhibition

**Figure 6:**
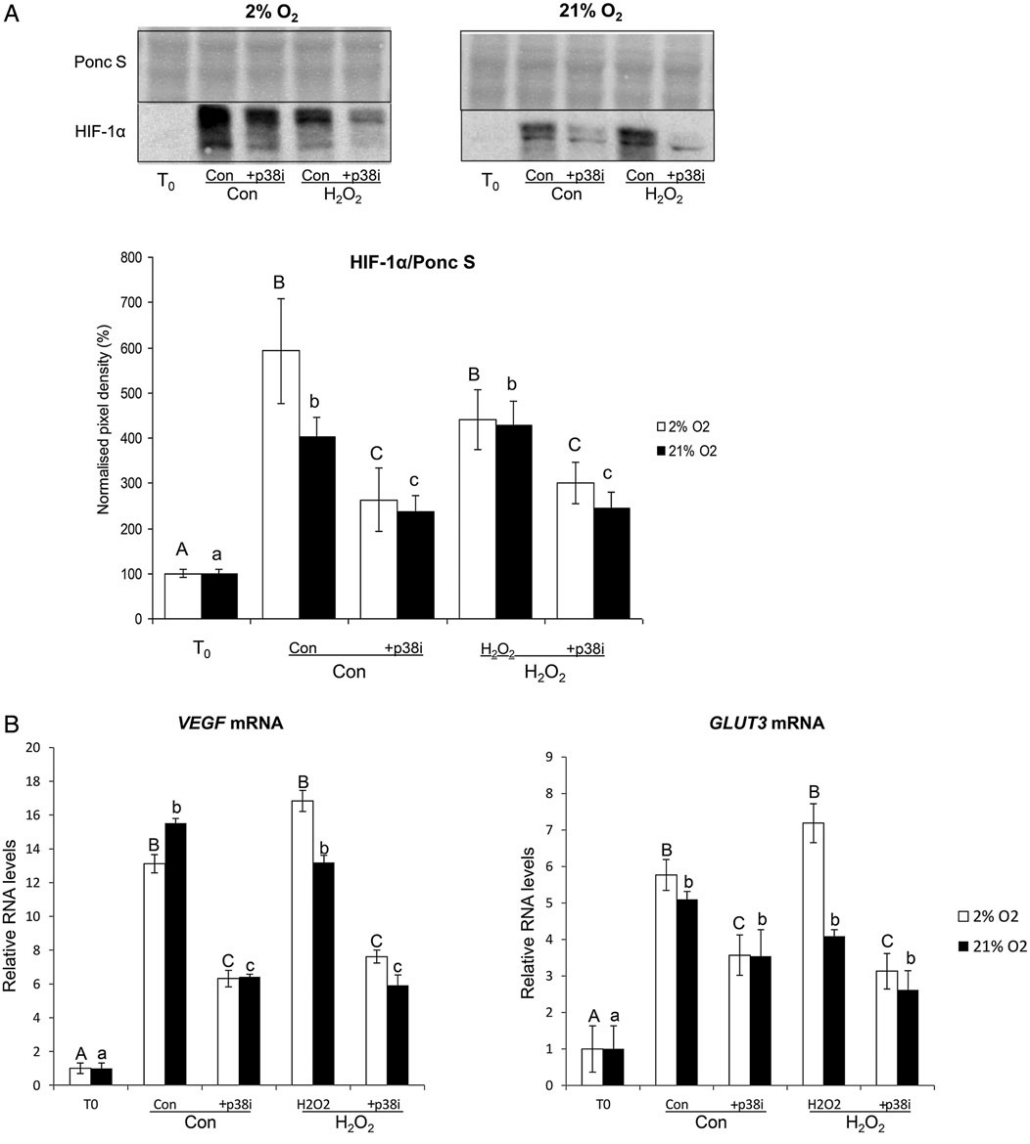
The effect of p38 inhibition on HIF-1α protein (**A**) and *VEGFA* and *GLUT3* mRNA (**B**) in first trimester placental explants subjected to 2 or 21% O_2_ ± 1 mM H_2_O_2_ for 6 h. (A) Lysates from first trimester explants cultured with or without the p38 inhibitor PD169316 (p38i; 10 µM) were immunoblotted with HIF-1α antibody. Poncaeu S staining served to normalize gel loading. Normalized results (±SEM) are plotted, expressing *T*_0_ samples as 100%. (B) RNA was isolated and relative levels of *VEGFA* and *GLUT3* mRNA were detected using quantitative real-time RT–PCR. *VEGFA* and *GLUT3* mRNA levels were normalized to the 18S RNA levels. Different letters indicate groups that are significantly different (*P* < 0.05; one-way ANOVA + Student–Newman–Keuls test).

Because of the increased p38 phosphorylation and multiple anti-HIF-1α immunoreactive bands in cultured samples, we hypothesized that p38 may regulate HIF-1α *in vitro*. Explants were pre-treated with a p38 inhibitor, PD169316 (10 µM), and subsequently cultured under 2 or 21% O_2_ ± 1 mM H_2_O_2_ (Fig. 6). Inhibition of the p38 pathway resulted in a significant reduction in HIF-1α in all samples (Fig. 6A), and suppression of *VEGFA* mRNA (Fig. 6B). PD169316 also reduced *GLUT3* mRNA, which is regulated by HIF-1α, but the suppression was only statistically significant in samples cultured under 2% O_2_ (Fig. 6B).

### Immunoprecipitation to evaluate the phosphorylation of HIF-1α

**Figure 7:**
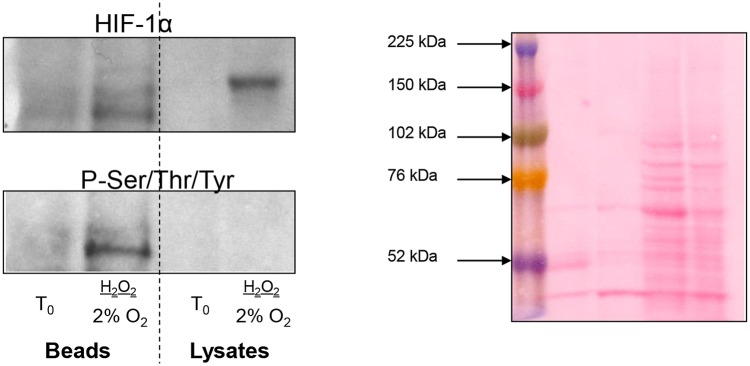
Immunoprecipitation experiment to evaluate the role of phosphorylation in HIF-1α regulation. Two samples from the same placenta (10 + 3 weeks) were snap frozen (T_0_) or cultured under 2% O_2_ in the presence of 1 mM H_2_O_2_ for 6 h. Lysates were immunoprecipitated with an antibody against phospho-Ser/Thr/Tyr, western blots were run, probed with anti-HIF-1α and re-probed with anti-phospho-Ser/Thr/Tyr. HIF-1α was only detected on the P-Ser/Thr/Tyr-coated beads from the cultured placenta.

Immunoprecipitation with an antibody against phospho-Ser/Thr/Tyr was performed on two samples from the same placenta (10 + 3 weeks) that were either snap frozen (*T*_0_) or cultured under 2% O_2_ in the presence of 1 mM H_2_O_2_ for 6 h. A western blot was probed with anti-HIF-1α and re-probed with anti-phospho-Ser/Thr/Tyr. Phosphorylated HIF-1α was only detected in the cultured placental samples, suggesting that phosphorylation could be involved in the up-regulation of its activity during culture (Fig. 7).

## Discussion

Because of the low intraplacental oxygen tensions reported, the first trimester intrauterine environment is frequently described as ‘hypoxic’. However, hypoxia cannot be defined by just the prevailing oxygen tension, but must be related to the metabolic demands of the tissue or cells. The term implies a pathologically low pO_2_ that is inadequate to meet the energy demands of the tissues, and so it is best evinced by metabolic parameters or transcriptional responses. Our finding that there were no differences in the ATP/ADP ratio, the levels of ADP, NAD^+^, lactate, or glucose across gestational ages indicate that the first trimester placenta is not energetically compromised. Thus, while the pO_2_ is low, it is inappropriate to describe this as ‘hypoxia’ as it is in fact the normal physiological state. The data also indicate that placental metabolism alters with gestational age, as the concentrations of PDE, Pcho and GPC, and taurine increased, whilst those of phosphomonoesters, polyols, hydrophobic amino acids (val/leu/ile) and creatine phosphate decreased towards term. We confirmed the decrease in creatine phosphate by showing a significant reduction in CK activity in term samples. Our findings complement the data of Thomure *et al.* (1996) who found reduced ubiquitous mitochondrial and cytosolic brain CK protein expression in term samples, compared with first and second trimester placentas. We also describe a remarkable increase in the PDE spectral intensity fraction and the PDE/PME spectral intensity ratio towards term. Whilst these parameters are unchanged between the first and second trimesters, there is a 15-fold increase in the PDE/PME ratio at term. Similar changes were reported by Sohlberg *et al.* (2014) in two groups of women with healthy pregnancies (mean gestational age 29.1 versus 37.9 weeks). The spectral intensity ratio of PDE/PME is regarded as an index of cell membrane turnover (Menon *et al.*, 1995), and the increase with gestational age could be explained by apoptosis (Smith *et al.*, 1997; Ishihara *et al.*, 2000) and a concomitant decrease in trophoblast cell proliferation (Ishihara *et al.*, 2000; Chen *et al.*, 2002).

The polyol metabolic pathway is highly active in the human conceptus during early pregnancy (Jauniaux *et al.*, 2005). This pathway will be advantageous under these conditions since it allows for regeneration of NAD^+^ to maintain glycolysis, and therefore ATP generation, without the need for oxygen. Although fewer molecules of ATP are produced per molecule of glucose than through oxidative phosphorylation, there is no evidence that these pathways are metabolically limiting if there is a plentiful supply of glucose. During the first trimester, the endometrial secretions are rich in glycogen, and this substrate accumulates in the syncytioplasm (Burton *et al.*, 2002), suggesting that this requirement is met.

The metabolomics study was a small-scale discovery analysis, using only 4–5 samples per group. We did not subject these results to correction for multiple testing because of the small numbers. Equally, the number of observations is too low to use principal component analysis (PCA). Thus, our interpretations of the data are based on statistical significance (*P* < 0.05) and the fact that concentrations of a variety of metabolites within specific pathways changed according to known biological pathways. Larger studies are therefore necessary to confirm our results.

In this study, neither HIF-1α nor HIF-2α were detectable in first trimester CVS samples, but could be detected in samples removed by curettage, the most common method of obtaining early placental tissue. Detectable HIF-1α and HIF-2α protein in the curettage samples could reflect oxidative stress, as these samples had been in contact with maternal blood. These samples showed increased p38 phosphorylation, which, as we show, likely plays a role in HIF-1α stabilization. Culture of unstressed first trimester samples under either 2 or 21% O_2_ for 6 h also induced a marked increase in HIF-1α and HIF-2α, oxidative stress and inflammation. Hydrogen peroxide, a pro-oxidant, had no additional effect, suggesting that the culture conditions *per se* were sufficient to induce the maximal stress responses. Other authors have reported an increase in HIF-1α protein (Caniggia *et al.*, 2000; Rajakumar and Conrad, 2000) and mRNA (Caniggia *et al.*, 2000) in first trimester samples cultured under 2% (Rajakumar and Conrad, 2000) or 3% (Caniggia *et al.*, 2000) O_2_ but not in samples cultured under what they describe as ‘normoxia’ (21% O_2_). Similar increase in HIF-2α protein was also reported in first trimester explants following 48 h incubation under 2% O_2_ (Genbacev *et al.*, 2001). The discrepancies with our results could be due to the starting material. It is not stated how the placental samples were obtained in these studies, whether they were collected immediately or were contaminated with maternal blood.

We previously showed that labour increases placental HIF-1α, VEGFA and sFlt1 mRNA and protein levels, but not PlGF levels (Cindrova-Davies *et al.*, 2007b). During labour, the placenta is exposed to repetitive ischaemia–reperfusion. Similarly, *in vitro* exposure of term placental villi to hypoxia-reoxygenation increased HIF-1α, sFlt1 and VEGFA protein levels. These effects could be blocked by administration of antioxidant vitamins, and by inhibiting the p38 MAPK and NF-κB pathways. Collectively, these findings suggest the involvement of oxidative stress signalling in HIF-1α and sFlt1 regulation (Cindrova-Davies *et al.*, 2007a; Cindrova-Davies, 2009). In this study, increased HIF-1α protein as well as associated up-regulation of *VEGFA* and *GLUT3* mRNA could also be blocked by inhibition of the p38 pathway. There was a marked mobility shift in the HIF in cultured explants, indicative of a phosphorylation regulation of this protein. Additionally, in our immunoprecipitation experiments, phosphorylated HIF-1α was detected in isolates of cultured placental tissue, but not of fresh tissue. This suggests that phosphorylation could be involved in its regulation and p38 is a likely effector. p38 can act as a pro-inflammatory kinase and the suppressive effects may be partly due to reduced levels of inflammatory markers. HIF-1α stability is increased by hypoxia, but it can also be up-regulated under non-hypoxic conditions by inflammatory cytokines or microtubule-depolymerizing agents involving the NF-κB pathway (Jung *et al.*, 2003a, b,). Our *in vitro* findings are consistent with reports that cytokine-mediated HIF-1α activation leads to production of VEGFA, and seems to proceed via a pathway involving an up-stream PI3K/AKT/mTOR pathway, NFκB activation and also COX2 expression (Jung *et al.*, 2003a, b).

The stability of HIF-1α and HIF-2α is regulated by prolyl hydroxylase domain (PHD) proteins. PHD hydroxylation of HIF-1α and HIF-2α under normoxia leads to HIF binding of VHL and subsequent polyubiquitination and proteosomal degradation (Maxwell *et al.*, 1999; Bruick and McKnight, 2001; Ivan *et al.*, 2001). Ietta *et al.* (2006) reported an inverse correlation between PHD proteins and HIF-1α in first trimester placentas, and demonstrated that degradation of HIF-1α takes place after 10 weeks of gestation, coincident with the placental O_2_ rise. Inhibition of PHDs activity increases HIF-1α stability in villus explants and stimulates TGFβ3 expression, which is important for placental development. PHD1, 2 and 3 (both protein and mRNA) are regulated by O_2_ in placental explants *in vitro*. In fact, low O_2_ (3%) induces mRNA of all three PHDs, compared with explants cultured under 20% O_2_. Similarly, exposure of explants to 3 and 8% O_2_ increases PHD1 and PHD3, but not PHD2 protein. The reduction in PHDs mRNA and protein under atmospheric O_2_ conditions could thus explain differences we see in HIF-1α expression between different starting materials.

In conclusion, there were no differences in the placental metabolomic profile across gestational age in terms of ATP/ADP ratio, NAD^+^, lactate, or glucose levels, indicating that the first trimester placenta is not compromised energetically. In addition, HIF-1α and HIF-2α protein were undetectable in first trimester villous samples collected by chorionic villus sampling. Culture of first trimester explants induced HIF-1α stabilization and up-regulation of the target genes, *VEGFA* and *GLUT3*, and down-regulation of *PlGF* under widely differing oxygen levels. Blocking the p38 MAPK pathway suppressed these changes. These data suggest that HIF-1α is unlikely to play an appreciable role in regulating placental transcriptional activity under steady-state conditions during the first trimester *in vivo*, but that the pathway may be activated by fluctuations in oxygenation.

## Authors’ roles

T.C.-D. planned and carried out most experiments and experimental analyses and wrote the first draft of the manuscript. M.T.P. carried out the metabolomics analysis and reviewed the manuscript. L.G. collected and sorted placental samples. E.J. obtained patient consent, obtained tissue under ultrasound guidance and critically reviewed the manuscript. G.J.B. provided experimental planning, data analysis and discussion and critically reviewed the manuscript. D.S.C.-J. provided experimental planning, data analysis and discussion and critically reviewed the manuscript.

## Funding

This work was supported by the Wellcome Trust (084804/2/08/Z). Funding to pay the Open Access publication charges for this article was provided by the Wellcome Trust.

## Conflict of interest

The authors have no conflict of interest.
